# Sequential drug release via chemical diffusion and physical barriers enabled by hollow multishelled structures

**DOI:** 10.1038/s41467-020-18177-2

**Published:** 2020-09-07

**Authors:** Decai Zhao, Nailiang Yang, Yan Wei, Quan Jin, Yanlei Wang, Hongyan He, Yang Yang, Bing Han, Suojiang Zhang, Dan Wang

**Affiliations:** 1 State Key Laboratory of Biochemical Engineering, Institute of Process Engineering, Chinese Academy of Sciences, No. 1 Beiertiao, Zhongguancun, 100190 Beijing, PR China; 2 University of Chinese Academy of Sciences, 19A Yuquan Road, 100049 Beijing, PR China; 3 Department of Geriatric Dentistry, NMPA Key Laboratory for Dental Materials, National Engineering Laboratory for Digital and Material Technology of Stomatology, Beijing Laboratory of Biomedical Materials, Peking University School and Hospital of Stomatology, Peking University, 100081 Beijing, PR China; 4 Key Laboratory of Green Process and Engineering, Institute of Process Engineering, Chinese Academy of Sciences, No. 1 Beiertiao, Zhongguancun, 100190 Beijing, PR China; 5 Department of Thoracic Surgery, Shanghai Pulmonary Hospital, Institute for Advanced Study, Tongji University, 200430 Shanghai, PR China; 6 Department of Orthodontics, Peking University School and Hospital of Stomatology, National Engineering Laboratory for Digital and Material Technology of Stomatology, and Beijing Key Laboratory of Digital Stomatology, Peking University, 22 Zhongguancun South Avenue, Haidian District, 100081 Beijing, PR China

**Keywords:** Drug delivery, Materials chemistry, Nanoscale materials

## Abstract

Hollow multishelled structures (HoMSs), with relatively isolated cavities and hierarchal pores in the shells, are structurally similar to cells. Functionally inspired by the different transmission forms in living cells, we studied the mass transport process in HoMSs in detail. In the present work, after introducing the antibacterial agent methylisothiazolinone (MIT) as model molecules into HoMSs, we discover three sequential release stages, i.e., burst release, sustained release and stimulus-responsive release, in one system. The triple-shelled structure can provide a long sterility period in a bacteria-rich environment that is nearly 8 times longer than that of the pure antimicrobial agent under the same conditions. More importantly, the HoMS system provides a smart responsive release mechanism that can be triggered by environmental changes. All these advantages could be attributed to chemical diffusion- and physical barrier-driven temporally-spatially ordered drug release, providing a route for the design of intelligent nanomaterials.

## Abstract (web summary)

Hollow multishell structures (HoMSs) consist of multiple shells with voids between them that provide separate reaction environments within the same assembly. Here, the authors used TiO2-HoMS to deliver a broad-spectrum antibacterial agent, in three-stages and in response to environmental changes.

## Introduction

Excellent bioinspired designs have long been desired by human society and have shown great advantages in many fields^1–6^. In the entities known as the building blocks of life, i.e., cells, various kinds of mass transport are essential to complex life activities. In addition to the common passive transmission through cell membranes caused by a concentration gradient, stimulus transmission is another typical property of cells allowing them adapt to the changing environment^7^. In this case, it is desired to design an artificial cell that can realize sequential mass release, including both passive and stimulus transmission. Emerging functional materials with hollow multishelled structures (HoMSs) can provide an abundant capacity for mass loading and benefit mass transport, thus enabling broad applications in solar cells, lithium-ion batteries, photocatalysis, etc.^8–14^. Importantly, HoMSs have a unique structure with multiple cavities and hierarchically porous shells, which excellently optimized the mass transport and effective facets exposure^15–19^. Our recent perspective has revealed that HoMSs have unique mass transport properties that strictly follow a temporal and spatial order during mass diffusion through the shells^20^. In this case, it can be predicted that using HoMS as a drug carrier for antibacterial agents will show some unexpected advantages. An ideal antibacterial system needs to meet the following requirements: (1) the rapid release of antibiotics to the environment at the concentration required for bacteriostatic treatment or sterilization; (2) maintenance of this concentration for a long time to prevent bacterial regrowth; and (3) autodetection of foreign pathogens and self-responsive release of the reserved antibiotics.

**Fig. 1:**
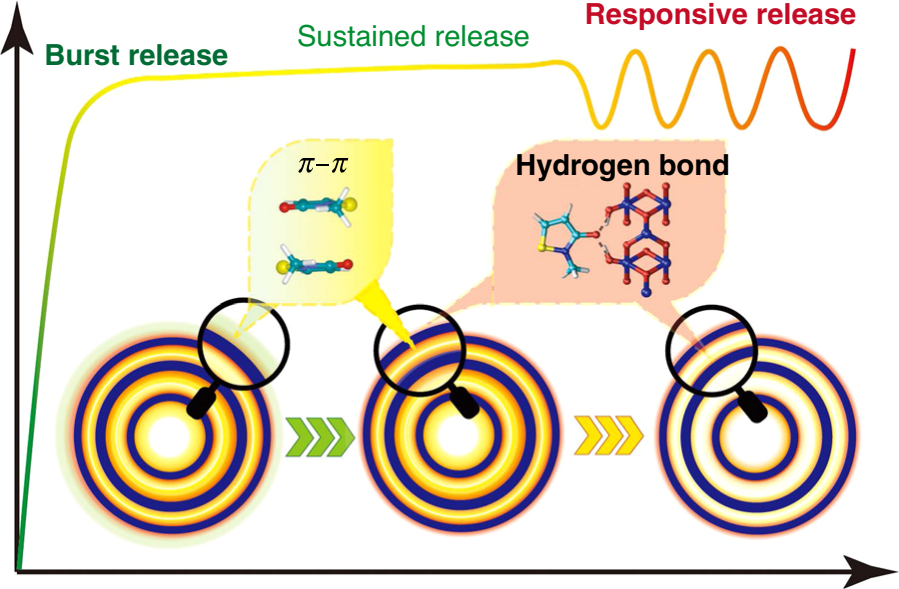
Schematic illustration of the spatially dependent drug release by HoMS. The green part represents drug molecules adsorbed on the outer surface of TiO_2_–HoMS, which are easily released at the initial stage, resulting in burst release; the yellow part represents drug molecules in the cavity, which interact through *π*–*π* interactions, which contribute not only to the sustained release stage but also more to the responsive release stage; the red part represents drug molecules that form hydrogen bonds with the TiO_2_ surface, which also contribute to part of the responsive release.

In our work, methylisothiazolinone (MIT) (Supplementary Fig. 1), a broad-spectrum antibacterial agent, is loaded into TiO_2_–HoMS to investigate its release properties, and the TiO_2_–HoMS is fabricated by the sequential templating approach (STA)^21^. Because MIT molecules can form hydrogen bonds with TiO_2_ and *π*–*π* stacking with each other^22^, and due to the capillary force in HoMS, notably, the sequential release, i.e., burst release, sustained release, and stimulus-responsive release, is realized in a single HoMS particle (Fig. 1), which is named as temporally, spatially ordered drug release.

## Results

### Characterization of MIT–TiO_2_–HoMS

**Fig. 2:**
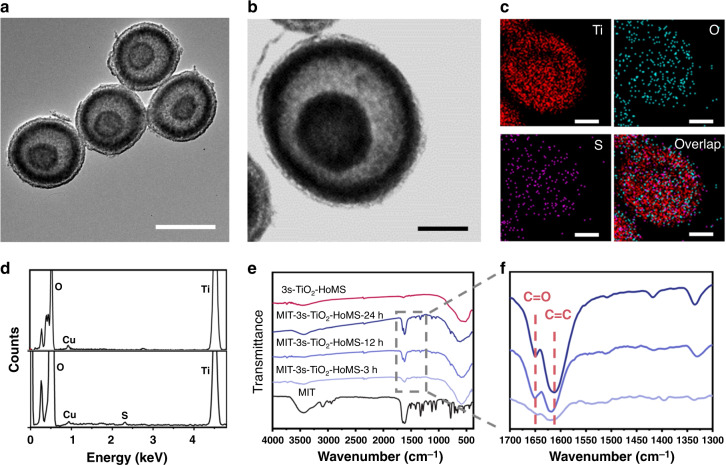
Characterization of TiO_2_–HoMS before and after MIT loading. **a** TEM image of 3s-TiO_2_–HoMS. The scale bar is 500 nm. **b** STEM image of MIT-3s-TiO_2_–HoMS. The scale bar is 200 nm. **c** TEM-EDX mapping images of MIT-3s-TiO_2_–HoMS. The scale bar is 200 nm. **d** TEM-EDX spectra of 3s-TiO_2_–HoMS (top) and MIT-3s-TiO_2_–HoMS (bottom). **e** FTIR spectra of MIT, 3s-TiO_2_–HoMS and MIT-3s-TiO_2_–HoMS with different drug-loading period. **f** Enlarged FTIR spectra in the selected region. Source data are provided as a Source Data file.

TiO_2_–HoMS samples with different shell numbers were fabricated through STA^20,23^ by adjusting the adsorption conditions of metal ions, and then MIT was loaded by a typical drug-loading process^24^. Transmission electron microscopy (TEM) images (Supplementary Fig. 2a, b and Fig. 2a) show that various samples with different shell numbers were fabricated. Based on the statistical analysis of more than 100 TiO_2_–HoMS particles for each sample, the average size of TiO_2_ hollow spheres and double-shelled (2s-) and triple-shelled (3s-) TiO_2_–HoMS is estimated to be 726 ± 47 nm, 642 ± 30 nm, and 583 ± 35 nm with a narrow size distribution, respectively (Supplementary Fig. 3). The average shell thickness and shell spacing are also given (Supplementary Table 1). The high-resolution TEM images demonstrate the high crystallinity and random distribution of the anatase and rutile TiO_2_ nanocrystals in the shells (Supplementary Fig. 2d)^12^. Furthermore, X-ray diffraction confirms that TiO_2_–HoMS is a composite of the anatase phase (JCPDS card No. 21-1272) and rutile phase (JCPDS card No. 21-1276) (Supplementary Fig. 2e)^25^. TEM-EDX mapping images of MIT-3s-TiO_2_–HoMS show that S is evenly distributed on the shell of TiO_2_–HoMS, indicating uniform drug loading (Fig. 2c, d). The cryo-TEM image shows a uniform contrast in HoMS after MIT loading, further indicating the successful loading of MIT (Supplementary Fig. 2f).

### MIT molecule absorption in HoMS

Fourier transform infrared (FTIR) spectra (Fig. 2e) were collected after different MIT loading periods to study the interaction between MIT and TiO_2_–HoMS. The peaks in the FTIR spectrum of MIT-3s-TiO_2_–HoMS at 714, 1418, 1618, and 1640 cm^−1^ could be ascribed to C–S (stretching), CH_3_ (bending), C=C, and C=O (stretching) in MIT^26^, respectively (Fig. 2e). Interestingly, a redshift is observed for the C=O stretching band with extension of the drug-loading period, indicating different adsorption modes during MIT loading (Fig. 2f).

**Fig. 3:**
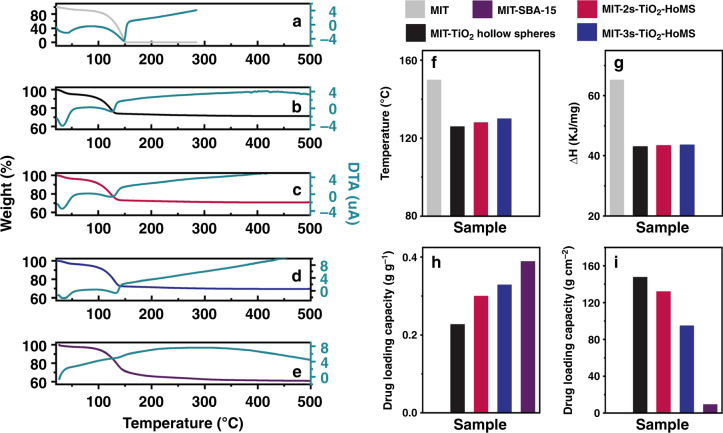
Adsorption, desorption, and diffusion of MIT molecules in HoMS. TG–DTA of **a** MIT, **b** MIT–TiO_2_ hollow spheres, **c** MIT-2s-TiO_2_–HoMS, **d** MIT-3s-TiO_2_–HoMS, **e** MIT-SBA-15. **f** Evaporation temperature and **g** relative value of endothermic heat for MIT release. MIT loading capacity after normalization to **h** weight and **i** surface area. Source data are provided as a Source Data file.

The thermal release of MIT through TiO_2_ hollow spheres and 2s- and 3s-TiO_2_–HoMS occurred in three stages, as exhibited in the thermogravimetric analysis (TGA) and differential thermal analysis (DTA) results (Fig. 3a–e). The first stage occurred at ~50 °C, with a weight loss of ~5% and accompanied by a endothermic process, which corresponded to the evaporation of water in this MIT–TiO_2_ system^27,28^. The second stage was in the range of 70–150 °C, which corresponded to the desorption of MIT loaded on the outside of the outer shells of HoMS and the MIT in cavities between shells. The third stage took place after 150 °C, and was associated with the disintegration of a small amount of MIT strongly bound to the shells of TiO_2_–HoMS by hydrogen bonds^29^. Notably, the second peak of the DTA curve of pure MIT was at 150 °C, while the peak temperature decreased to 126, 127, and 130 °C after MIT was loaded into the TiO_2_ hollow spheres and 2s- and 3s-TiO_2_–HoMS, respectively (Fig. 3f). Furthermore, according to the Speil theory^30^, the relative value of the endothermic heat can be calculated by integrating the DTA curve and then normalizing to the mass of MIT (Fig. 3g). The relative values are 65.28, 43.01, 43.38, and 43.57 kJ mg^−1^ for pure MIT, MIT–TiO_2_ hollow spheres and MIT-2s- and MIT-3s-TiO_2_–HoMS, respectively. It can be noted that after loading MIT into HoMS, a smaller driving force is needed for molecule release. The MIT loading capacity can be calculated as 0.2274, 0.3000, and 0.3292 (normalized to the weight of TiO_2_) for TiO_2_ hollow spheres and 2s- and 3s-TiO_2_–HoMS, respectively. This result indicates that building up more surface in a single hollow particle is helpful for drug adsorption. In this case, an increased number of shells improved the drug-loading capacity, proving that HoMS is a good candidate for mass loading. Notably, when we used SBA-15 as the carrier, the MIT loading amount was calculated as 0.3888 after normalization to the weight of SiO_2_, which is higher than the values obtained using TiO_2_ as a carrier (Fig. 3h). However, the specific surface area loading capacity (Fig. 3i) of 3s-TiO_2_–HoMS is surprisingly 46.5 times higher than that of SBA-15 owing to the much larger effective surface area of HoMS (Supplementary Table 2). To investigate the type of absorption between MIT and TiO_2_–HoMS, the Langmuir (first equation) and Freundlich isothermal adsorption models (second equation) were used to fit the experimental data^31^:

1 $$\frac{1}{{q_e}} = \frac{1}{{Q_0}} + \frac{1}{{Q_0K_Lc_e}},$$

where *c*_*e*_ = equilibrium concentration of adsorbate (mg L^−1^), *q*_*e*_ = amount of MIT adsorbed per gram of carrier at equilibrium (mg g^−1^), *Q*_0_ = maximum monolayer coverage capacity (mg g^−1^), and *K*_*L*_ = Langmuir isotherm constant (L mg^−1^):

2 $$lg\,q_e = lg\,K_f + \frac{1}{n}lg\,c_e,$$

where *K*_*f*_ = Freundlich isotherm constant (mg g^−1^), *n* = adsorption intensity, *c*_*e*_ = equilibrium concentration of adsorbate (mg L^−1^), and *q*_*e*_ = amount of MIT adsorbed per gram of carrier at equilibrium (mg g^−1^). The R^2^ values of the Langmuir model and the Freundlich model for MIT adsorption on TiO_2_–HoMS are 0.3685 and 0.9931, respectively (Supplementary Fig. 4). Therefore, the adsorption of MIT on TiO_2_–HoMS can be considered as multimolecular layer adsorption with Freundlich model.

### Sequential drug release

**Fig. 4:**
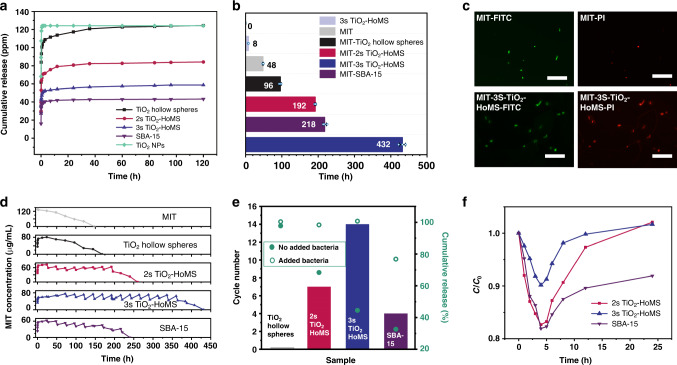
MIT release and antibacterial performance. **a** Cumulative release performance of MIT in buffer with the same amount of MIT in different carriers. **b** Sterile maintenance performance under different conditions with the same MIT amount. All error bars are equivalent (SD positive and negative values) and represent standard deviation with *n* = 3. **c** Corresponding fluorescence microscopy images of *E. coli*. The presented fluorescence images were taken on the 10th day of the microbial strength tests of MIT and MIT-3s-TiO_2_–HoMS, which started with the same MIT amount. The scale bar is 5 μm. The merged images were processed and analyzed by ImageJ software. **d** Bacterial-responsive release profiles of MIT, MIT–TiO_2_ hollow spheres, MIT-2s-TiO_2_–HoMS, MIT-3s-TiO_2_–HoMS, and SBA-15. **e** Antibacterial cycling performance with introduced bacteria (columns) and cumulative release percentage with bacteria (hollow circles) and without bacteria (solid circles). **f** Responsive release performance of different carriers during one antibacterial stimulation cycle. Source data are provided as a Source Data file.

Taking the release of MIT, a model antibacterial compound, for characterization purposes, the mass release performance was tracked by UV–Vis spectrophotometer (Supplementary Fig. 5). The MIT release process shows roughly three stages (Fig. 4a). After the MIT-carrier composite entered the solution, a quick increase in drug concentration was observed at the first stage (~4 h), which is called burst release. Afterwards, the mass release speed slowed, and the MIT concentration remained stable at the second stage, which is named sustained release. 2s- and 3s-TiO_2_–HoMS and SBA-15 all have the capacity to maintain the concentration in the second stage (Fig. 4a). Notably, when carrying the same drug amount, 3s-TiO_2_–HoMS was found to be the most effective in inhibiting the growth of *Escherichia coli* (*E. coli*) when we continually introduced bacteria into the system and could inhibit bacterial growth even after 432 h (Fig. 4b). The presented fluorescence images^32^ show that on the 10th day, the bacterial viability was 66%, 58%, 33%, 0%, and 11% for MIT, MIT-loaded TiO_2_ hollow spheres, MIT-2s-TiO_2_–HoMS, MIT-3s-TiO_2_–HoMS, and MIT-SBA-15, respectively (Fig. 4c and Supplementary Fig. 6). Moreover, all the TiO_2_ carriers show good stability, which did not present any morphological changes even after 30 days (Supplementary Fig. 7).

Interestingly, this stimulus-responsive release, the third stage of sequential release, is a unique feature of the HoMS system. After the concentration of MIT stabilized at ~60 ppm (slightly higher than the minimum inhibitory concentration (MIC)) with the same total drug amount for various samples (Supplementary Fig. 8), bacteria were added to the solution to investigate the responsive release performance. 2s- and 3s-TiO_2_–HoMS and SBA-15 all presented responsive release performance, i.e., after the rapid decrease in the concentration of MIT, equilibrium was gradually recovered. 3s-TiO_2_–HoMS showed the best recycling performance among all the samples and maintained the concentration over the MIC after 14 rounds of addition of bacteria. Impressively, the recovery process was different for various samples. 3s-HoMS showed a smaller drop (9.8%) than 2s-HoMS (17.3%) with the same amount of bacteria added (Fig. 4f), indicating that the MIT molecules reserved in 3s-HoMS are easier to release under the stimulating conditions. In comparison, the MIT-loaded TiO_2_ hollow spheres and pure MIT do not show the ability of responsive release (Fig. 4e). MIT-loaded SBA-15 has excellent sustained-release performance; however, its responsive release is not as good as that of 3s-TiO_2_–HoMS. SBA-15 shows the largest drop of 18.1% after adding bacteria (Fig. 4f), and it cannot reach the initial concentration during the recovery stage, even after 20 h.

In addition to responding to bacteria, MIT–HoMS can also respond to pH changes (Supplementary Fig. 9a). A low pH value can change the zeta potential of TiO_2_ from −19.96 to 0.42 mV, weaken TiO_2_–MIT interactions and further affect MIT–MIT interactions; thus, more MIT molecules can be easily released (Supplementary Fig. 9b).

## Discussion

A key parameter for sequential mass release is the interaction between chemical molecules and carriers. In this MIT–TiO_2_–HoMS system, spatially, there are three different molecular loading sites. The first site is the outermost surface of HoMS. The second is the cavities between shells, where molecules exist via *π*–*π* stacking (Supplementary Fig. 10). The third loading site is the inner surface of HoMS, where the interactions between the molecules and TiO_2_ involve hydrogen bonding.

At the initial stage of drug loading, the drugs diffuse into HoMS by capillary force. According to the equation used to calculate the liquid height induced by capillary force, *h* = 2*σ*cos*θ*/(*ρgr*). Except for the capillary radius *r*, all the other parameters (*σ* is the surface tension coefficient, *θ* is contact angle, *ρ* is liquid density, and *g* is gravitational acceleration) are the same in our system. This means that the driving force is inversely proportional to the capillary radius, so decreasing the shell spacing of the HoMS can increase the capillary force. As shown, 3s-TiO_2_–HoMS has the smallest distance between the outermost and middle shells (Supplementary Table 1), and it should have the largest capillary force for drug absorption. The absorbed drugs will first attach to the surface of the shell by hydrogen bonds. The hydrogen bonds between C=O groups and TiO_2_ weakened C=O bonds and caused a redshift in the FTIR spectrum (Fig. 2e). The hydrogen bonds formed between S atoms and TiO_2_ weakened C–S bonds, thus inducing the adjacent C=C bonds to be stronger, and a blueshift in C=C bonds was observed. As the MIT loading period is gradually extended to 24 h, drug molecule uptake through *π*–*π* stacking dominates, and the infrared spectrum of MIT–HoMS increasingly resembles that of pure MIT. The different molecular loading sites will cause temporally ordered mass release.

During the first stage of MIT release, the MIT molecules attached on the outside surface of HoMS immediately spread into the medium because the concentration gradient is large at the initial stage and there is no shell barrier for the outside molecules.

The following second stage, i.e., sustained release, is beneficial to simultaneously kill bacteria and prevent the regrowth of bacteria for a long time^33,34^. At this point, the concentration gradient still provides the driving force. Notably, different from the burst release, the molecules stacked by *π*–*π* interactions and stabilized by capillary forces in the cavities between shells contribute more for this stage. These molecules could be slowly released to the environment through the hierarchical pores on the shells (Supplementary Fig. 11). By combination with the physical barriers introduced by the multi-shell configuration, the release period is prolonged^19,32,35–37^. It is worth noting that the concentration plateau for 3s-TiO_2_–HoMS is lower than that of other TiO_2_ systems with the same amount of drug added, which means that the drug molecules are more difficult to release from the triple shells. This phenomenon can also be observed from the TG–DTA results (Fig. 3f, g), where greater energy consumption is necessary for 3s-TiO_2_–HoMS during MIT evaporation. As in the previous analysis, 3s-TiO_2_–HoMS would provide a stronger capillary force than 2s-TiO_2_–HoMS and the hollow spheres, resulting in greater resistance to release from 3s-TiO_2_–HoMS. In conclusion, this long period of sustained release reflects the balance between the driving force (concentration gradient) and transmission resistance (hydrogen bonding, *π*–*π* stacking, and capillary force). In other words, a stronger driving force is necessary to break the balance, which can induce the third stage of release, i.e., stimulus-responsive release.

Interestingly, when the driving force is strong enough to break the energy barrier, rapid release is achieved in the HoMS system. This responsive release is dominated by the drug molecules stored between the shells (by *π*–*π* stacking) and absorbed on the surface (by hydrogen bonding) of HoMS, and the drug concentration can be recovered to the desired range automatically. It can be observed from the TG–DTA results (Fig. 3f, g), that less heat consumption is necessary for drug evaporation through HoMS, indicating that the cavity and nanopores of HoMS can promote mass release after the driving force requirement is met. Moreover, pH changes and foreign pathogens can also be used to trigger molecular release, thus broadening the applications of this system. In comparison, although mesoporous SBA-15 has excellent sustained-release performance, its responsive release is not as good as that of the HoMS system (Fig. 4f). The long channels in SBA-15 are not favorable for mass transport, leading to a long recovery period after stimulus. In this case, the third stage of sequential release, i.e., stimulus-responsive release, is a unique property of the HoMS system.

In summary, cell-like TiO_2_–HoMS was successfully synthesized and used as a smart drug carrier. By using MIT, a common antibacterial agent, as the model molecule, superb sustained release was obtained, and the corresponding antibacterial duration of the MIT-3s-HoMS system was nearly eight times longer than that of pure MIT without HoMS under the same conditions. The relatively isolated cavities and hierarchical pores in HoMS result in different chemical and physical environments in space, which induce various forms of drug loading and temporally ordered drug release. Importantly, a smart and efficient stimulus-responsive release is obtained that can be triggered by replenishing foreign pathogens. This is the first report to discover the sequential release consisted with three release stages together in a single HoMS particle, which provides a route for the design of intelligent nanomaterials in the near future.

## Methods

### Materials

The reagents including sucrose, ethanol, and titanium tetrachloride were of analytical grade, and purchased from the Beijing Chemical Reagent Factory without further purification. MIT (95%) was purchased from Aldrich. *E. coli* JM109 were purchased from Tiangen Biochemical Technology Co., Ltd.

### Synthesis of HoMS TiO_2_

The carbonaceous microspheres template was obtained by emulsion polymerization reaction of sucrose under hydrothermal conditions^25^. Typically, 130 g of sucrose was dissolved in 250 mL of deionized (DI) water and sealed into a 500 mL Teflon reactor followed by reaction at 200 °C for 132 min. The brown product was washed six times with DI water and ethanol, and then dried at 80 °C for 12 h.

0.6 g of the as-prepared carbonaceous microspheres was dispersed in 30 mL of 3 mol L^−1^ TiCl_4_ aqueous solution under ultrasonication for 20 min. The resulting suspension was aged for a certain time in the room temperature or a 40 °C water bath under stirring followed by filtration, washing twice with DI water, and drying at 80 °C for 12 h. The resultant composite was heated to 500 °C in air at a heating rate of 2 °C min^−1^ and held for 3 h, and a white powdered product was obtained. The specific preparation condition of the HoMS TiO_2_ is listed in Supplementary Table 3.

### Drug loading

MIT was selected as a guest drug molecule for loading. Steps were as follows: first, 8 mg of the HoMS TiO_2_ was placed in a sealed container, and then the air in the TiO_2_ cavity was eliminated with a vacuum system. Second, 2 mL solution of MIT 10 wt% MIT was added rapidly, and then the mixture was stirred at room temperature for 24 h. Finally, the MIT-loaded HoMS TiO_2_ was separated by centrifugation (1844 × *g*, 5 min) to wash off the free MIT and then the products were dried under vacuum at 40 °C overnight. All of these operations were in the dark condition. Several different MIT concentrations have been tested. To ensure a high drug-loading capacity and encapsulation efficiency, we chose the concentration at the intersection of the two curves (Supplementary Fig. 12) as the concentration of the MIT.

### Drug release

The release of MIT from HoMS TiO_2_ in a disodium hydrogen phosphate-citric acid buffer solution was followed using UV–Vis spectroscopy. MIT-loaded HoMS TiO_2_ with the same MIT amount were dispersed in the buffer solution at room temperature and then transferred into a dialysis bag (Mw cut-off:14,000 Da). The bag was placed into 50 mL buffer solution. At predetermined intervals, 10 μL of the suspension was taken out from the mixture, and the absorbance of MIT in the clear upper liquid was analyzed by UV–Vis spectroscopy at a wavelength of 273 nm. This step was repeated in every interval until the release of MIT reached equilibrium.

### Bacterial-responsive release

MIT-loaded HoMS TiO_2_ with the same MIT amount were dispersed in the buffer solution at room temperature and then transferred into a dialysis bag (Mw cut-off: 14,000 Da). The bag was placed into 50 mL buffer solution. When the release reached equilibrium, 200 µL *E. coli* was dropped in to make the concentration of *E. coli* was 10^6^ CFU mL^−1^. 10 µL of suspension was taken out and diluted ten times after adding bacteria for 4 and 24 h, and the concentration was measured with UV–Vis spectrophotometer. Bacteria were added every 24 h. The equilibrium concentration of MIT was controlled around 60 ppm, which shows a higher bacterial inhibition efficiency (Supplementary Fig. 13).

### MIC test

Serial twofold dilutions of MIT ranging from 3.125 to 400 μg mL^−1^ were prepared in a 96-well microtiter plate using LB medium. Freshly grown *E. coli* bacteria were inoculated into each cell to reach a final concentration of 10^6^ CFU mL^−1^ (refer to the bacterial concentration specified in the antimicrobial sensitivity test, established by Clinical and Laboratory Standards Institute). After those cells were incubated for 24 h at 37 °C, bacterial growth in each cell was monitored and compared with that of the positive-control cell to which no MIT was added. The MIC was recorded as the lowest concentration of MIT that completely inhibited bacterial growth in 24 h, which is 50 ppm.

### Bacterial viability test

To observe the antibacterial activity of the MIT-loaded HoMS TiO_2_, *E. coli* were grown in 4 mL LB medium and supplemented with MIT-loaded HoMS TiO_2_ with 0.6 mg MIT. 100 μL of the bacterial suspension was pipetted into EP tube and then stained with fluorochrome followed by fluorescence observation in every 24 h, and then supplemented with a 100 μL fresh bacterial solution.

For optical imaging, *E. coli* bacteria were stained by fluorescein isothiocyanate (FITC) for 1 h and propidium iodide (PI) solution for 5 min. After washing three times with PBS solution (pH = 7.4), fluorescence microscope (Olympus IX73) was used to image the bacterial samples. All living and dead bacteria appear green at an excitation wavelength of 488 nm by FITC staining, whereas only dead cells appear red at an excitation wavelength of 543 nm with PI staining. All of the bacterial experiments were repeated at least three times to give an average value.

### Analytical methods

TEM images were obtained on a FEI Tecnai F20 instrument operated at an accelerating voltage of 200 kV. Cryo-TEM samples were prepared at FEI Vitrobot Mark IV. Scanning electron microscopy images were obtained using a JSM-7001F microscope operating at 15.0 kV voltages. Field TGA–DTA was performed on a SDTA DSC851e thermo analysis instrument in nitrogen atmosphere. UV–visible absorption spectra of various samples were obtained by a Shimadzu UV-1780 UV–Vis spectrophotometer with the scanning region of 200–700 nm. The nitrogen adsorption–desorption isotherms were measured on a Quantochrome Autosorb-1MP sorption analyzer under liquid nitrogen (−196 °C) with prior degassing under vacuum at 200 °C for more than 12 h. The FTIR patterns were performed through a T27-Hyperion-Vector22 (Bruker). Fluorescent microscopic images were taken on an Olympus IX73.

### Statistics and reproducibility

All the results can be repeated from at least three independent experiments.

### Reporting summary

Further information on research design is available in the Nature Research Reporting Summary linked to this article.

## Supplementary information

Supplementary Information

Reporting Summary

## Data Availability

The data underlying Figs. 2, 3a, and 4e as well as Supplementary Figs. 2, 4, 5, 6, 7, and 8 are available in the associated source data file. All other data supporting the findings of this study are available within the paper and its supplementary information files. Source data are provided as a Source Data file by figshare (https://figshare.com/s/b0068c6bb47c67e446df).
