# Supplementary material for: Sequential drug release via chemical diffusion and physical barriers enabled by hollow multishelled structures
Source: Nat Commun. 2020 Sep 7;11:4450. doi: 10.1038/s41467-020-18177-2 (PMC7477205; doi:10.1038/s41467-020-18177-2)
Supplement: Supplementary file 1 — Supplementary Information [file 41467_2020_18177_MOESM1_ESM.pdf]

Supplementary Information

Sequential drug release via chemical diffusion and physical barriers enabled by hollow multishelled structures

Zhao et al.

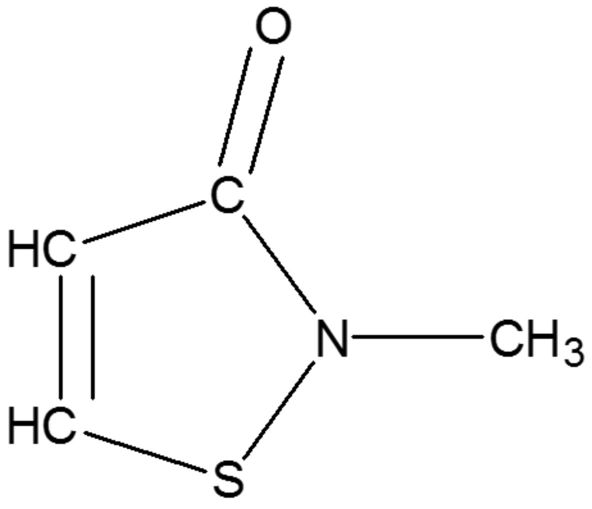

Supplementary Figure 1 Molecular structure of Methylisothiazolinone (MIT).

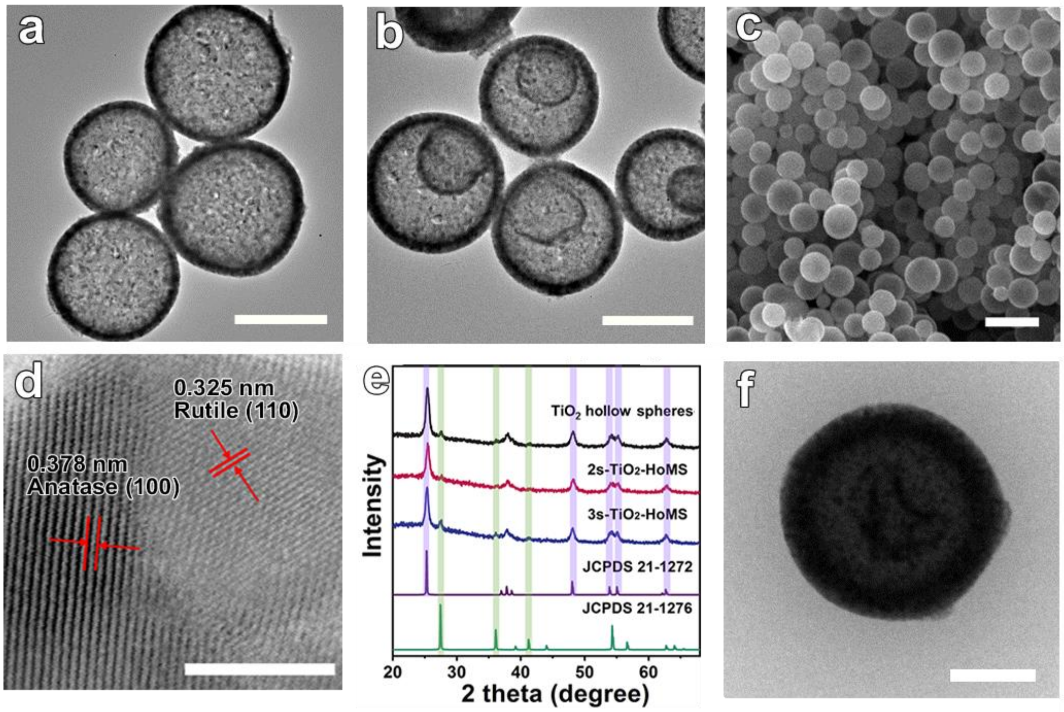

Supplementary Figure 2 Morphology and crystal structure characterization of TiO_2 hollow structures. TEM images of (**a**) TiO_2 hollow spheres and (**b**) 2s-TiO_2-HoMS.The scale bar is 500 nm. (**c**) SEM image of 3s-TiO_2-HoMS. The scale bar is 1 μm. (**d**) HRTEM image of 3s-TiO_2-HoMS. The scale bar is 5 μm. (**e**) XRD pattern of 3s-TiO_2-HoMS. (**f**) Cryo-TEM image of MIT-2s-TiO_2-HoMS.The scale bar is 200 nm.

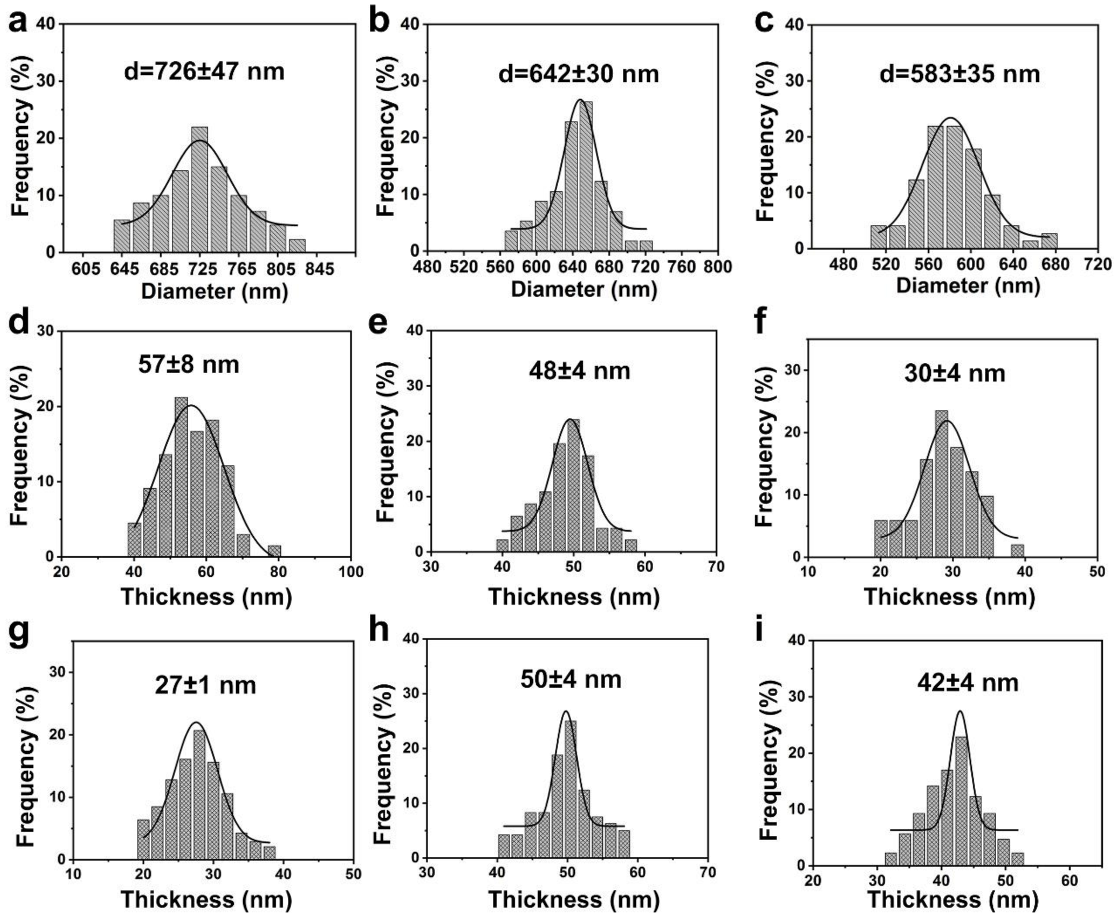

Supplementary Figure 3 Particle size statistic histograms of (**a**) TiO_2 hollow spheres, (**b**) 2s-TiO_2-HoMS and (**c**) 3s-TiO_2-HoMS. (**d**) The shells thickness distribution of TiO_2 hollow spheres. (**e**) Outermost and (**f**) innermost shell thickness distribution of 2s-TiO_2-HoMS. (**g**) Outermost, (**h**) middle and (**i**) innermost shell thickness distribution of 3s-TiO_2-HoMS (n=100 TiO_2-HoMS).

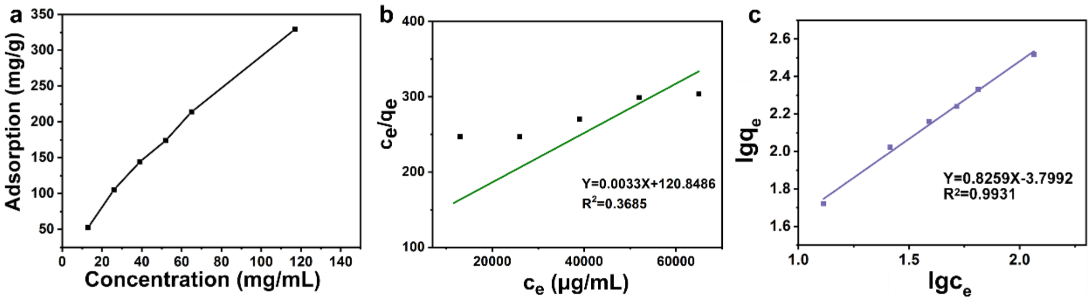

Supplementary Figure 4 (**a**) MIT adsorption curve of 3s-TiO_2-HoMS versus the initial concentration of MIT in buffer solution. (**b**) The linear relationship between c_e and c_e/q_e fitted by Langmuir isothermal adsorption model. (**c**) The linear relationship between lgc_e and lgq_e fitted by Freundlich isothermal adsorption model. c_e equals to the equilibrium concentration of MIT, and q_e equals to equilibrium adsorption quantity (mg MIT per gram adsorbent).

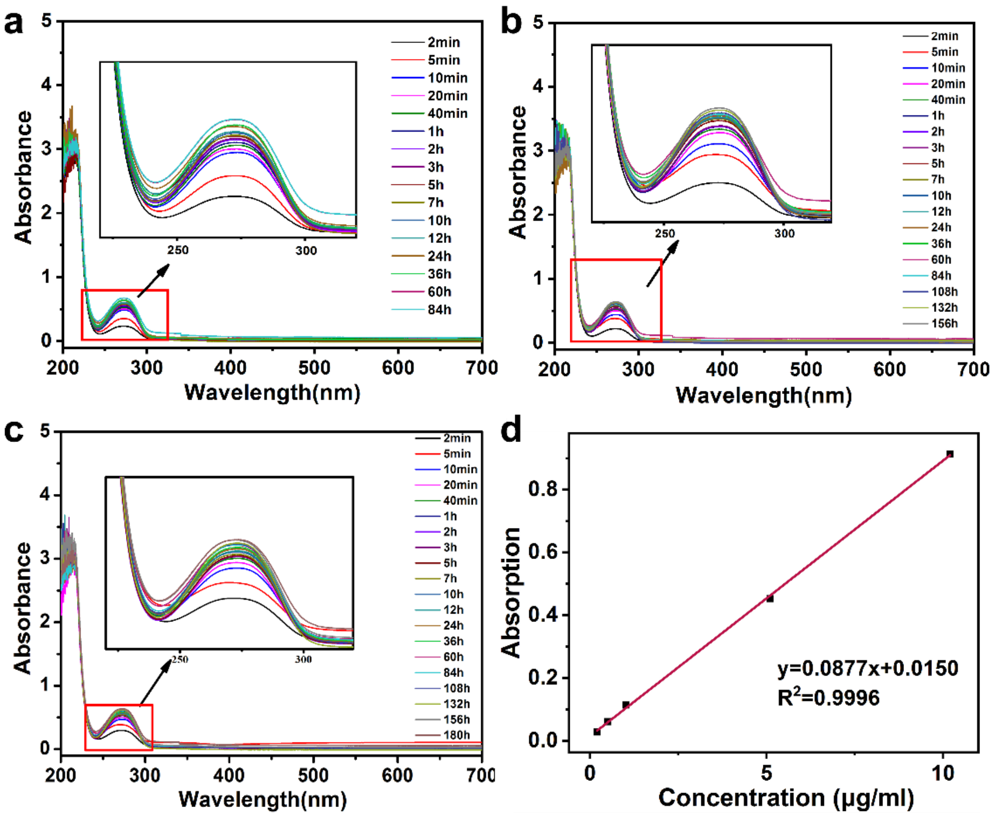

Supplementary Figure 5 UV-vis absorption spectroscopy of MIT released by (**a**) MIT-TiO_2 hollow spheres, (**b**) MIT-2s-TiO_2-HoMS, and (**c**) MIT-3s-TiO_2-HoMS in pH=7 buffer solution. (**d**) Linear relationship between absorbance at 278 nm *vs.* MIT concentration. Solid line is the liner fit with R^2 = 0.9996.

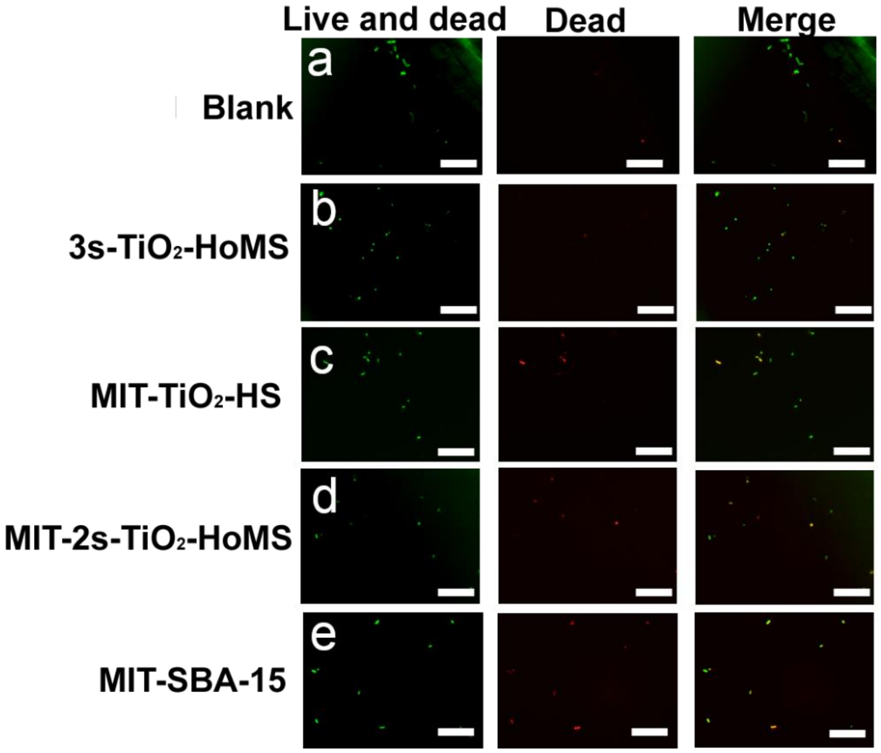

Supplementary Figure 6 Fluorescence images of microbial strength test at the 10th day and bacterial viability was 100%, 100%, 58%, 33%, and 11% for (a) blank (no MIT, no TiO_2), (b) 3s-TiO_2-HoMS (no drug), (c) MIT-TiO_2 hollow spheres, (d) MIT-2s-TiO_2-HoMS and (e) MIT-SBA-15, respectively. The drug adding amount was 0.6 mg, which is the same for MIT-TiO_2 hollow spheres, MIT-2s-TiO_2-HoMS and MIT-SBA-15. 10^6 CFU/mL of *E.coli* was added every 24 hours. Green: *E.coli* stained with FITC at an excitation wavelength of 488 nm in fluorescence microscopy to present both the live and dead ones. Red: *E.coli* stained with PI at an excitation wavelength of 543 nm in fluorescence microscopy to represent the dead ones. Yellow: the merge of green and red. The scale bar is 5 μm.

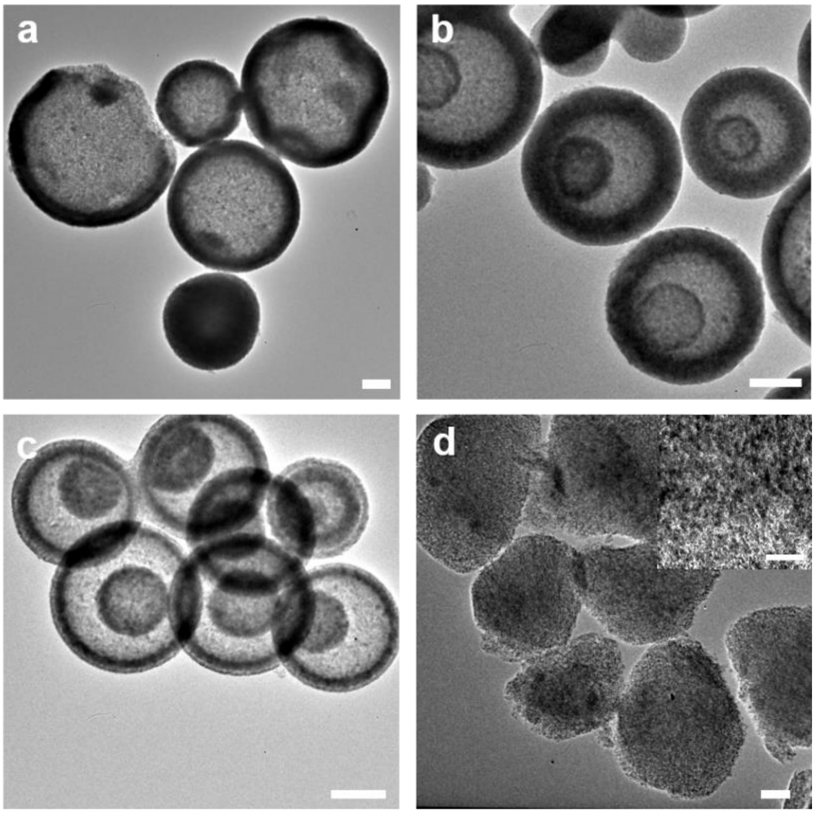

Supplementary Figure 7 TEM images of (a)TiO_2 hollow spheres, (b) 2s-TiO_2-HoMS, (c) 3s-TiO_2-HoMS, and (d) SBA-15 after MIT releasing for 720 h. The scale bar is 200 nm. (inset: enlarged image of SBA-15, scale bar: 100 nm).

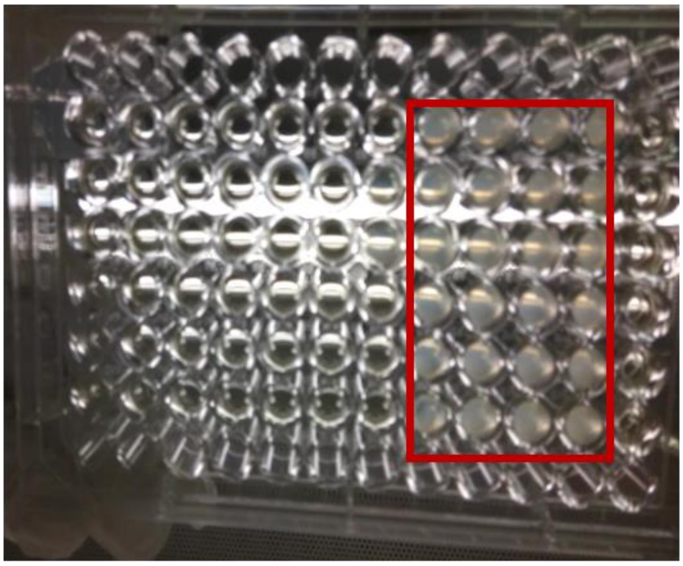

Supplementary Figure 8 Pictures of 96-well plates after cultivated for 24h. The MIC of MIT was 50 ppm for *E.coli*. Bacteria in well plates surrounded by red circles are not inhibited under the MIT concentration of 3.125, 6.25, 12.5, 25 ppm, respectively (from right to left).

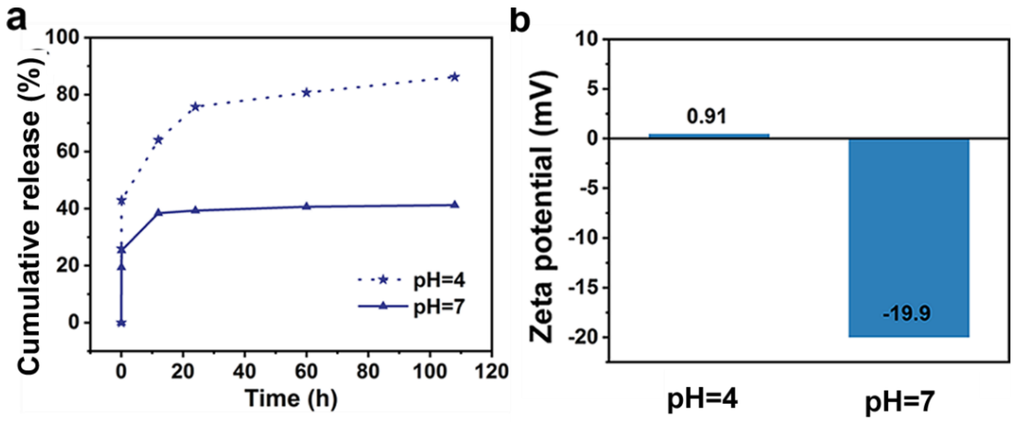

Supplementary Figure 9 (**a**) Cumulative release percentage of MIT from 3s-TiO_2-HoMS in the buffer solution with pH=4 (dot line) and 7 (solid line) for 120 hours. (**b**) Zeta potential of TiO_2-HoMS under the condition of pH=4 and 7, respectively.

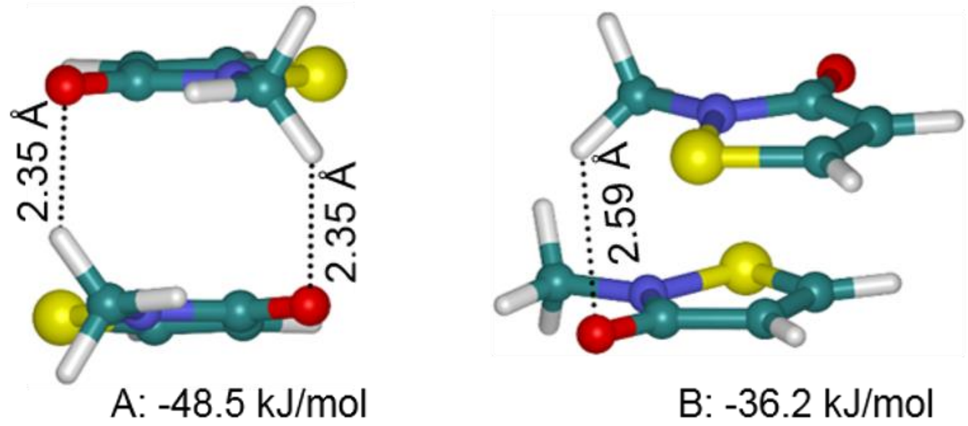

Supplementary Figure 10 The interaction energy by π-π stacking for the different configuration of the MIT-MIT system from the MD simulations.

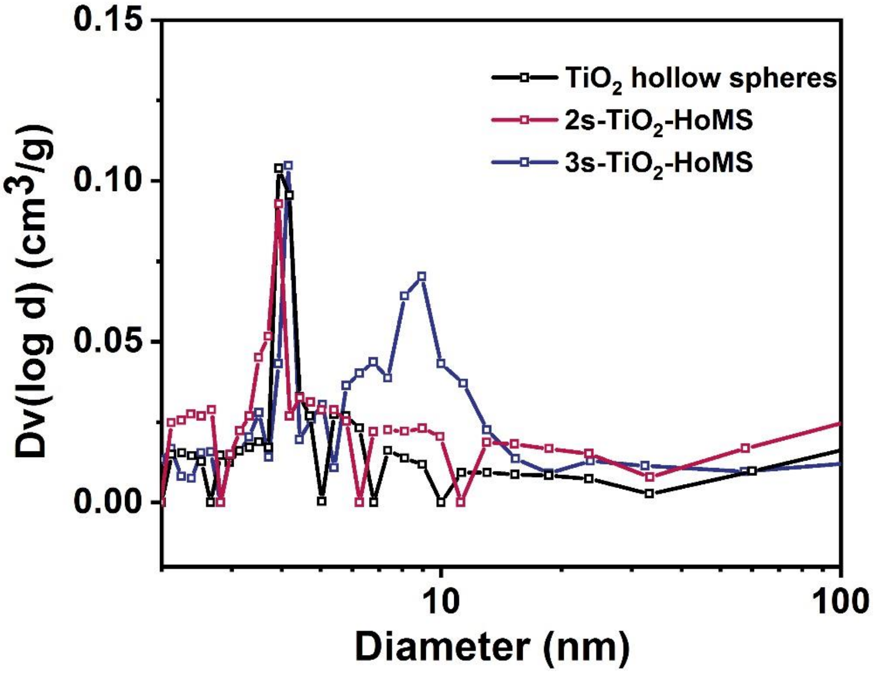

Supplementary Figure 11 Distribution of pore diameters of different TiO_2 carriers. These curves indicate the hierarchical pores in the samples.

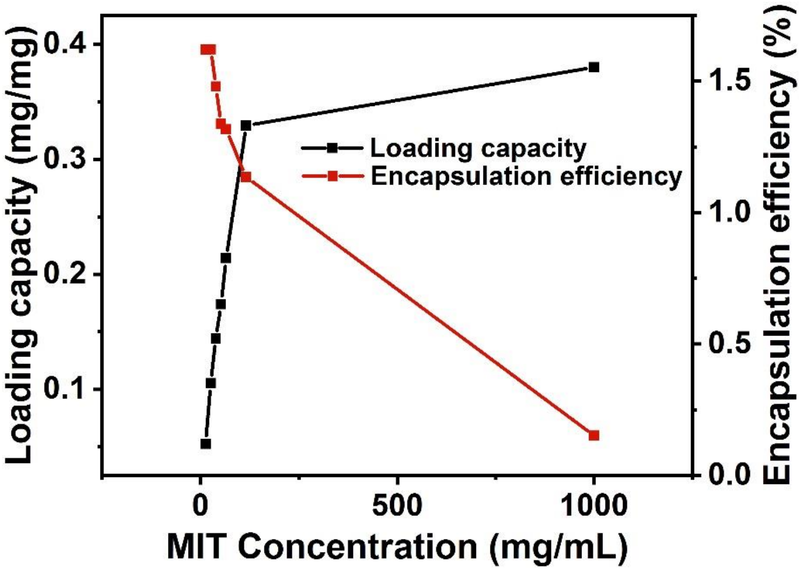

Supplementary Figure 12 MIT loading capacity (black curve) and entrapment efficiency (red curve) of 3s-TiO_2-HoMS. To ensure a high drug loading capacity and encapsulation efficiency, we chose the concentration of the intersection of the two lines as the concentration of the MIT loading.

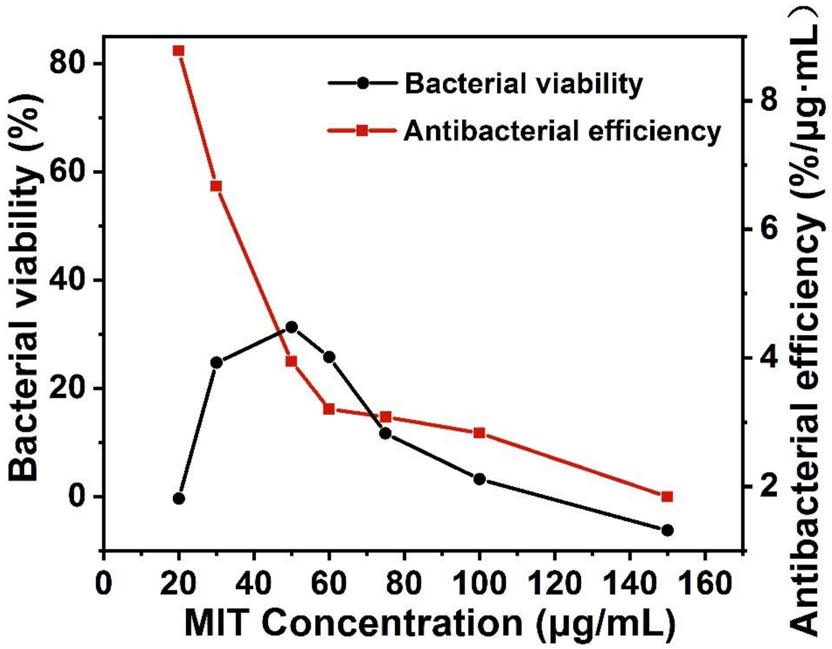

Supplementary Figure 13 Bacterial viability and antibacterial efficiency under different MIT concentration.

Supplementary Table 1 The average particle size, shell thickness, and shell spacing of TiO_2 hollow spheres, 2s- and 3s-TiO_2-HoMS.

Morphology	Diameter/nm	Shell thickness/nm	Shell spacing/nm
TiO_2 hollow spheres	726	57	—
2s-TiO_2-HoMS	642	48 (outermost)<br>30 (innermost)	79
3s-TiO_2-HoMS	583	28 (outermost)<br>50 (middle)<br>42 (innermost)	21<br>92

Supplementary Table 2 Specific surface area and loading capacity of TiO_2 hollow spheres, TiO_2-HoMS, TiO_2 NPs, and SBA-15 for drug delivery system.

Carrier	Specific surface area (m^2/g)	Loading capacity (g MIT/g carrier)	Loading capacity (g MIT/cm^2 carrier)
TiO_2 hollow spheres	15.4	0.2274	147.6
2s-TiO_2-HoMS	22.7	0.3000	132.1
3s-TiO_2-HoMS	34.65	0.3292	95
TiO_2 NPs	380.0	0.0951	9.5
SBA-15	409	0.3888	2.5

Supplementary Table 3 Fabrication conditions for different TiO_2 carriers. 3 mol/L $TiCl_4$ aqueous solution is adopted for the adsorption of carbonaceous microsphere.

Morphology	Adsorption conditions	Calcination process
TiO_2 hollow spheres	6 h, 25 ºC	2 °C min^{-1} to 500 °C, held for 3 h
2s-TiO_2-HoMS	8 h, 40 ºC	
3s-TiO_2-HoMS	24 h, 40 ºC	
